# Multiple sclerosis can be diagnosed solely with dissemination in space: Yes

**DOI:** 10.1177/13524585241245313

**Published:** 2024-04-14

**Authors:** Ahmed T Toosy, Frederik Barkhof

**Affiliations:** NMR Research Unit, Queen Square MS Centre, Department of Neuroinflammation, UCL Queen Square Institute of Neurology, Faculty of Brain Sciences, University College London, London, UK; NMR Research Unit, Queen Square MS Centre, Department of Neuroinflammation, UCL Queen Square Institute of Neurology, Faculty of Brain Sciences, University College London, London, UK; Centre for Medical Imaging Computing, Department of Medical Physics and Biomedical Engineering, Faculty of Engineering Science, University College London, London, UK; Department of Radiology and Nuclear Medicine, Amsterdam UMC, Vrije Universiteit Amsterdam, Amsterdam, The Netherlands

Currently, the diagnosis of multiple sclerosis (MS) requires two conditions: dissemination in space (DIS) and dissemination in time (DIT), but only after a demyelinating clinical episode. DIT is evidenced by a second demyelinating event, either clinical or radiological, although within the current 2017 diagnostic criteria, cerebrospinal (CSF)-specific oligoclonal bands (OCBs), may substitute for DIT.^1^

We propose that DIS on magnetic resonance imaging (MRI) alone is sufficient to diagnose MS, without the requirement for DIT or (further) clinical events. This is predicated upon two assertions – the first supports the notion of DIT redundancy and the second recognises pathological consistency across multiple MS phenotypes. There is also greater confidence in radiologically identifying inflammatory demyelination and excluding MS mimics.

Historically, DIT was included with DIS in the Poser criteria of 1983, during an era of non-MRI paraclinical tests.^2^ It was duly incorporated into the original McDonald criteria^3^ and has persisted as a necessary condition, purportedly to ensure diagnostic specificity. However, immunopathogenic knowledge of MS lesions has increased over time and histological identification of a specific pathology should not depend on DIT, for example, the histological diagnosis of a specific neoplasm does not depend on its evolution or whether it is symptomatic. MRI can identify and distinguish MS from non-MS lesions based on central nervous system (CNS) topography and morphological characteristics^4^ and this distinction is not DIT dependent; other CNS white matter conditions such as cerebral small vessel disease also exhibit DIT. Indeed, it is not realistically possible to distinguish between MS and non-MS aetiologies when a new deep white matter lesion appears in a patient suspected of MS, yet it can currently count as DIT.

Contrary to the clinical situation, on MRI, the findings of DIT and DIS are conceptually inter-related: when identifying lesions affecting multiple compartments, it is extremely unlikely that all lesions appeared simultaneously to fulfil DIS. The development of acute clinically isolated syndrome (CIS) in a patient with DIS, showing non-enhancing – and therefore older – lesions itself signifies that DIT is already fulfilled. This argument strengthens with a higher number of lesions or compartments involved on MRI. Furthermore, classical DIT requires a second focal demyelinating event after a symptomatic first one. However, disease-modifying treatments (DMTs) are increasingly aimed towards achieving no evidence of disease activity – even in CIS. This ideology appears to controvert the 2017 McDonald criteria, for example, radiological or clinical DIT may theoretically never be achieved after a CIS with DIS- and CSF-specific OCBs, following high efficacy treatment. Finally, improving specificity by incorporating DIT is unnecessary, because the 2017 diagnostic criteria are applied after excluding better explanations for the clinical presentation. This pre-condition will enhance specificity of the criteria in favour of attaining a genuine MS diagnosis and is not DIT dependent. The arguments presented above potentially render DIT redundant in diagnosing MS. The fact that it still exists is likely to represent a legacy from the original Poser clinical diagnostic criteria.

Second, there is acceptance that different MS-related phenotypes, including radiologically isolated syndromes (RIS) and primary progressive multiple sclerosis (PPMS), are underpinned by common pathobiological mechanisms. Recently, DMTs have delayed time to the first clinical event in RIS (dimethyl fumarate^5^ and teriflunomide^6^) just as beta-interferons delayed time to second events in CIS in earlier treatment studies. RIS patients share similar normal-appearing brain MRI metrics with CIS/MS^7^ and MR lesional characteristics should be indistinguishable from MS. Risk factors for disease activity and progression are identical between RIS and CIS, for example, younger age, CSF-OCBs, infratentorial and spinal cord lesions.^8^ Finally, asymptomatic demyelination at post-mortem and brain biopsies in RIS are histologically identical to MS.^9^ For PPMS, there is evidence that it represents the more neurodegenerative end of a pathophysiological spectrum seen in MS. Hence, RIS, CIS and other MS phenotypes can be unified under one pathological entity.

We propose that the identification of multiple focal CNS lesions on MRI, consistent with inflammatory demyelination by DIS topography and morphology, should be sufficient to diagnose MS. If MRI criteria for DIT are omitted in certain cases, it would be prudent to consider modified DIS criteria. Other considerations include (1) obtaining evidence-based international consensus opinion to determine in whom DIT requirement could be omitted (e.g. if already three or four compartments affected); (2) developing an iterative process to include CIS with only DIS then, later, RIS; (3) CSF-OCBs may help to support DIS although DIS with negative CSF-OCBs still exhibit very high risk of converting to 2010 McDonald MS;^10^ interestingly, CSF-OCBs were historically included as supporting DIS in the original McDonald criteria^3^ before being removed, then returned in the 2017 revisions, and this is most likely because their presence has prognostic value in predicting future activity, hence their versatility in support of DIS or as a substitute for DIT; (4) incorporation of MRI acquisitions to identify cortical/subpial involvement, chronic active lesions and central vein signs; (5) considering evidence for other topographical combinations such as multiple short segment cord lesions (which, in isolation, might be sufficient to diagnose MS) and (6) re-evaluation of the original Poser criteria if insufficient radiological evidence to support clinical DIS. After diagnosing MS based on DIS, features such as DIT, gadolinium enhancement and other paraclinical tests, for example, neurofilament levels, could contribute to informing disease prognosis and treatment decisions, also crucial in MS management.

There are caveats and limitations though. DIS criteria may need to be adjusted for lower specificity situations such as older populations at greater risk of small vessel vasculopathy, although DIT is still not specific in this situation. ADEM-like presentations with the appearance of multi-focal, near-simultaneous lesions need consideration. The proposition above is biased towards the diagnosis of relapsing-remitting MS, but it should be possible to incorporate PPMS into this diagnostic framework.

While the call for a DIS-based diagnostic framework may appear revolutionary, it actually represents an iterative evolution of the MS diagnostic criteria, as techniques that improve pathological accuracy continue to be developed and validated into the 21st century. DIT would still be important in stratifying activity and prognosis to inform treatment decisions rather than being necessary for diagnosis.
